# Supplementary material for: Brain function and metabolism in patients with long-term tacrolimus therapy after kidney transplantation in comparison to patients after liver transplantation
Source: PLoS One. 2020 Mar 10;15(3):e0229759. doi: 10.1371/journal.pone.0229759 (PMC7064204; doi:10.1371/journal.pone.0229759)
Supplement: S1 Table — TE: echo time; TI: Inversion time; TR: repetition time. (DOCX) [file pone.0229759.s002.docx]

Supplemental Table 1

| Sequence | Field of view (mm^2^) | Pixel size (mm^2^) | Slice thickness (mm) | Resolution  (mm) | TR (ms) | TE  (ms) | TI (ms) | Flip angle |
| --- | --- | --- | --- | --- | --- | --- | --- | --- |
| T2 weighted turbo spin echo sequence (TSE) with triple echos | 256x208 | 1x1 | 3 | 1 | 6440 | 8.7/70/131 | - | 150° |
| T2*-weighted gradient-echo sequence (GRE) with triple echos | 256x208 | 1x1 | 3 | 1 | 1410 | 6/18/30 | - | 20° |
| T1-weighted 3D Magnetization Prepared Rapid Gradient Echo (MPRAGE) | 256x224 | 1x1 | 1 | 1 | 1900 | 2.93 | 900 | 9° |
| T2-weighted-fluid-attenuated inversion recovery sequence (FLAIR) | 230x194 | 1x0.9 | 5 | 1 | 9000 | 94 | 2500 | 150° |
