# Supplementary figures and images for: Brain function and metabolism in patients with long-term tacrolimus therapy after kidney transplantation in comparison to patients after liver transplantation
Source: PLoS One. 2020 Mar 10;15(3):e0229759. doi: 10.1371/journal.pone.0229759 (PMC7064204; doi:10.1371/journal.pone.0229759)

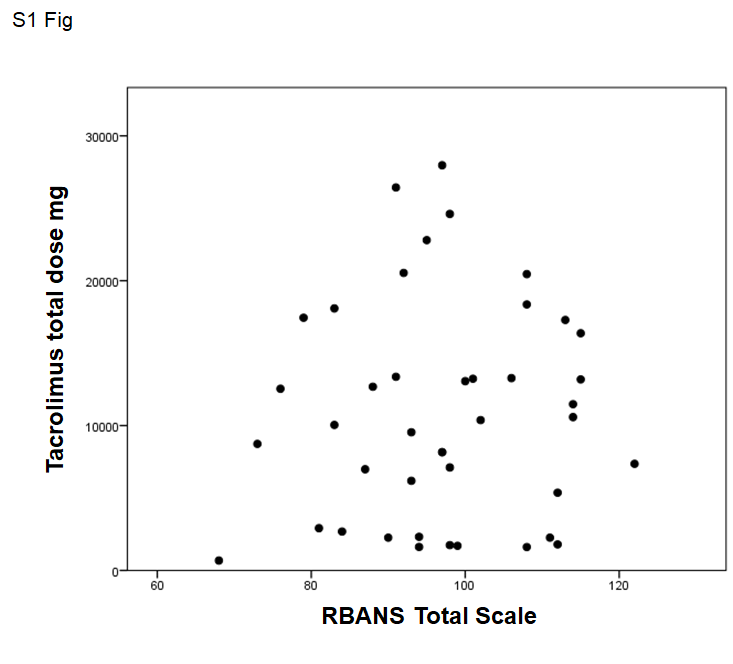

Supplement: S1 Fig — r = 0.62, p = 0.69; RBANS: Repeatable Battery for the Assessement of Neuropsychological Status. (TIF) [file pone.0229759.s003.tif]

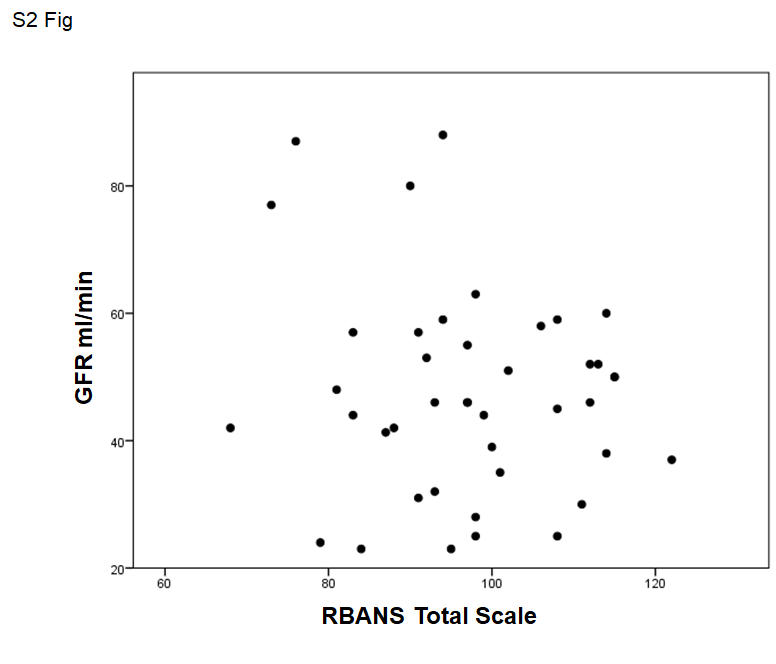

Supplement: S2 Fig — r = -0.15, p = 0.35; RBANS: Repeatable Battery for the Assessement of Neuropsychological Status; GFR: glomerular filtration rate. (TIF) [file pone.0229759.s004.tif]

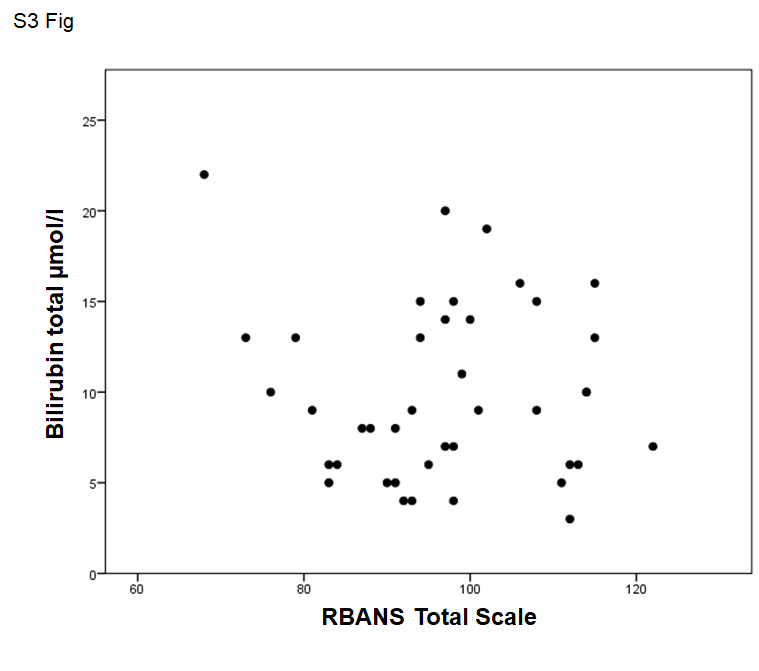

Supplement: S3 Fig — r = -0.76, p = 0.64; RBANS: Repeatable Battery for the Assessement of Neuropsychological Status. (TIF) [file pone.0229759.s005.tif]

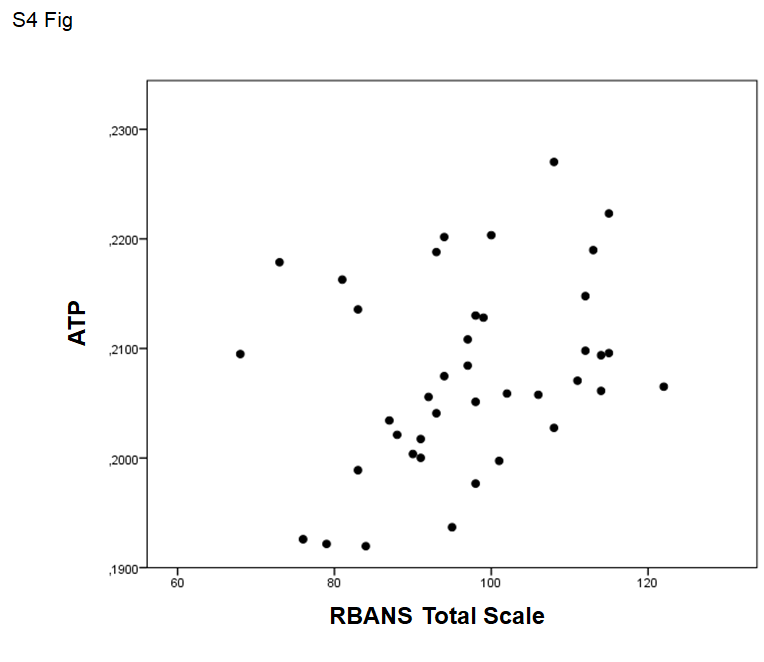

Supplement: S4 Fig — r = 0.31, p = 0.06; RBANS: Repeatable Battery for the Assessement of Neuropsychological Status; ATP: Adenosine triphosphate. (TIF) [file pone.0229759.s006.tif]

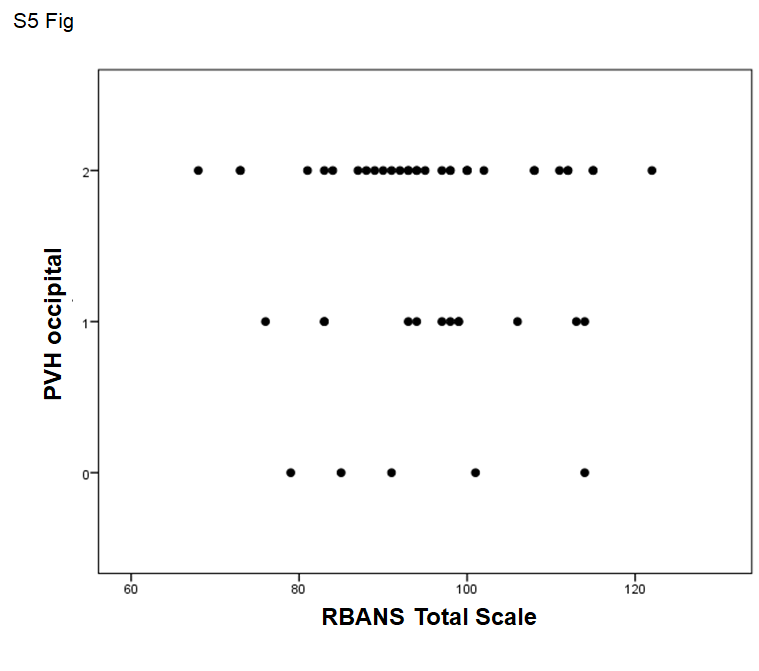

Supplement: S5 Fig — r = 0.002, p = 0.99; RBANS: Repeatable Battery for the Assessement of Neuropsychological Status; PVH: periventricular hyperintensities. (TIF) [file pone.0229759.s007.tif]
